# Bilateral choroidal tuberculoma in miliary tuberculosis - report of a case

**DOI:** 10.1186/s12348-014-0032-x

**Published:** 2015-02-13

**Authors:** Radha Annamalai, Jyotirmay Biswas

**Affiliations:** Sri Ramachandra University, Porur, Chennai, 600166 India; Uveitis & Ocular Pathology Department, Sankara Nethralaya, 18, College Road, Nungambakkam, Chennai, 600006 India

**Keywords:** Choroidal tuberculoma, *Mycobacterium tuberculosis*, Real-time polymerase chain reaction, Aqueous humor, Angiography

## Abstract

**Background:**

A choroidal mass or granuloma is a feature of ocular tuberculosis (TB). Tubercles can arise in the early stages of progression of TB and indicate hematogenous dissemination before the development of symptomatic disease. Tubercular subretinal granuloma is responsive to treatment with anti-tuberculosis therapy, and prompt treatment helps resolve the lesion completely.

**Findings:**

We report a case of a solitary large active choroidal tuberculoma in one eye and multiple healed tubercles in the other. The patient was an immunocompetent girl with splenic and miliary TB and had a relatively asymptomatic systemic status. Aqueous humor analysis by polymerase chain reaction (PCR) and real-time PCR (RT-PCR) was negative for the *Mycobacterium tuberculosis* genome. Based on clinical history and investigations, treatment with four-drug regimen of anti-tubercular treatment with concomitant corticosteroids was started, and total healing of the lesion occurred in 3 months.

**Conclusions:**

This case highlights the fact that in some patients, analysis of the aqueous may not provide any clue to the confirmation of an active tubercular choroidal granuloma and an association between splenic tuberculosis and choroidal tubercles has never been reported in the past.

## Findings

### Introduction

The ocular involvement of tuberculosis is not uncommon but diverse. The ophthalmic presentation of ocular tuberculosis might be caused by direct infection or indirect immune-mediated hypersensitivity. There is an increasing incidence of tuberculosis even in the developed countries, due to migration from endemic areas and increase in the number of immunocompromised patients [1]. Ocular tuberculosis can involve any tissue of the eyes and the orbit. The rich vascularity of uveal tissues permits easy hematogenous spread of infections from a caseous primary focus elsewhere in the body by eroding blood vessels or lymphatics. Tubercles can arise in the early stages of progression of TB and indicate hematogenous dissemination before the development of symptomatic ocular tuberculosis (TB) [2]. Tubercular uveitis can exist even in the absence of systemic disease and could be due to hypersensitivity that develops towards the tubercle DNA. We report a case of choroidal tuberculoma in a patient with miliary tuberculosis and the features noted on imaging studies performed on this patient which include fundus fluorescein angiography (FFA), indocyanine green angiography (ICG), and optical coherence tomography (OCT).

### Case report

**Figure 1:**
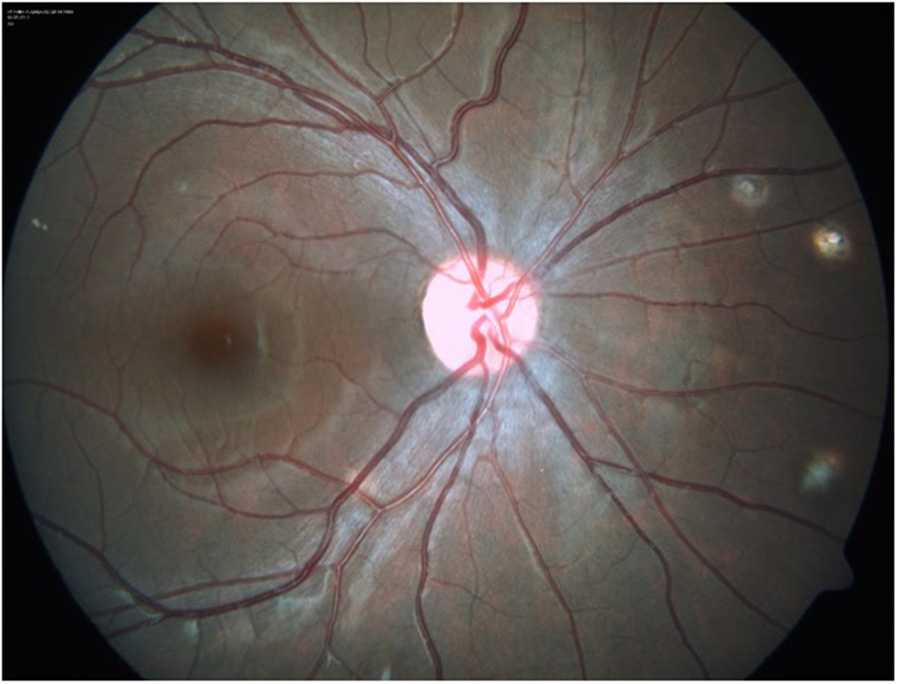
**Color fundus photo of the right eye showing multiple healed choroidal tuberculomas.**

**Figure 2:**
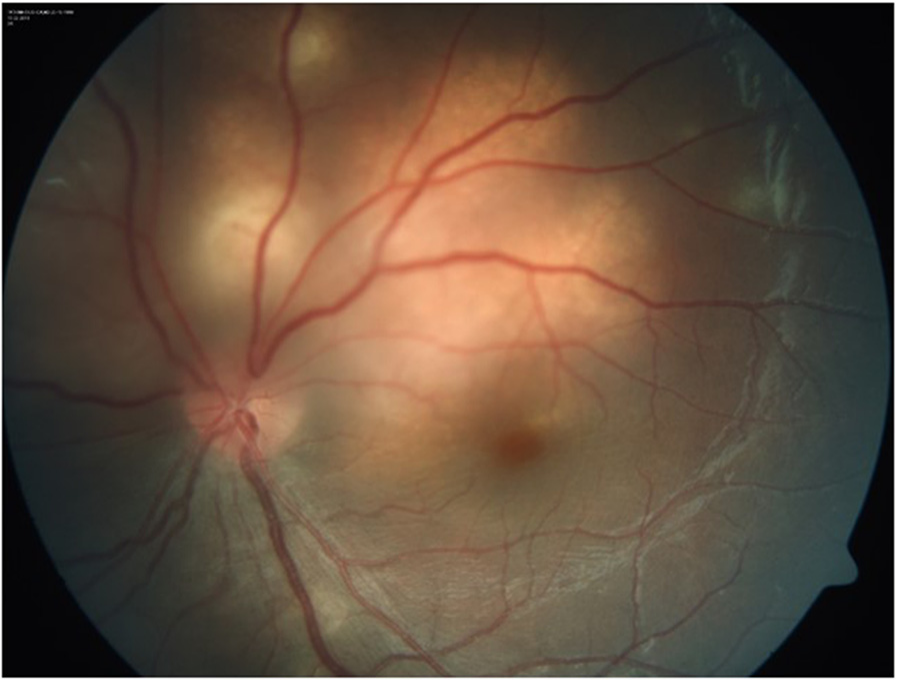
**Color fundus photo of the left eye showing an active tuberculoma of the choroid.**

**Figure 3:**
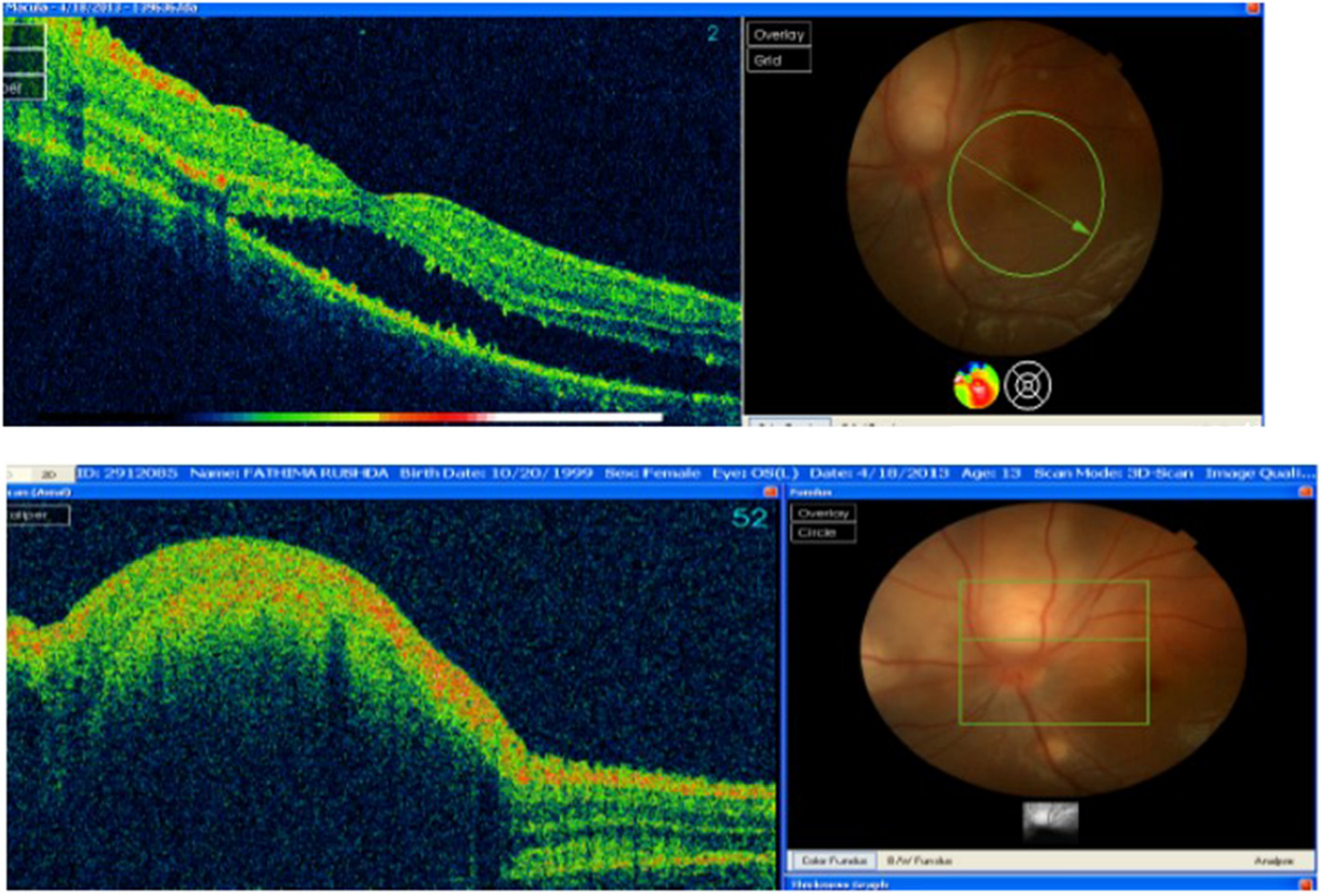
**OCT - left eye showing marked serous detachment of neurosensory retina superior and superotemporal to disc.** (Involvement of macula is seen)

**Figure 4:**
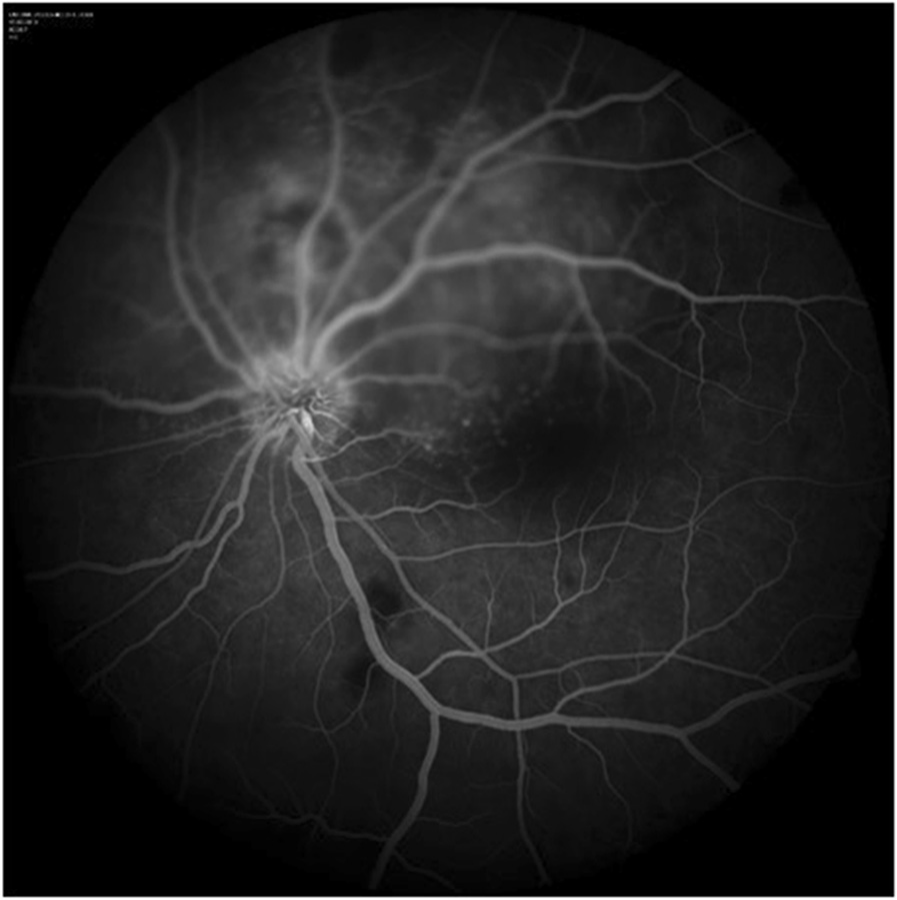
**Early arteriovenous phase of fluorescein angiogram showing diffuse hyperfluorescence due to subretinal leakage of dye in the left eye.**

**Figure 5:**
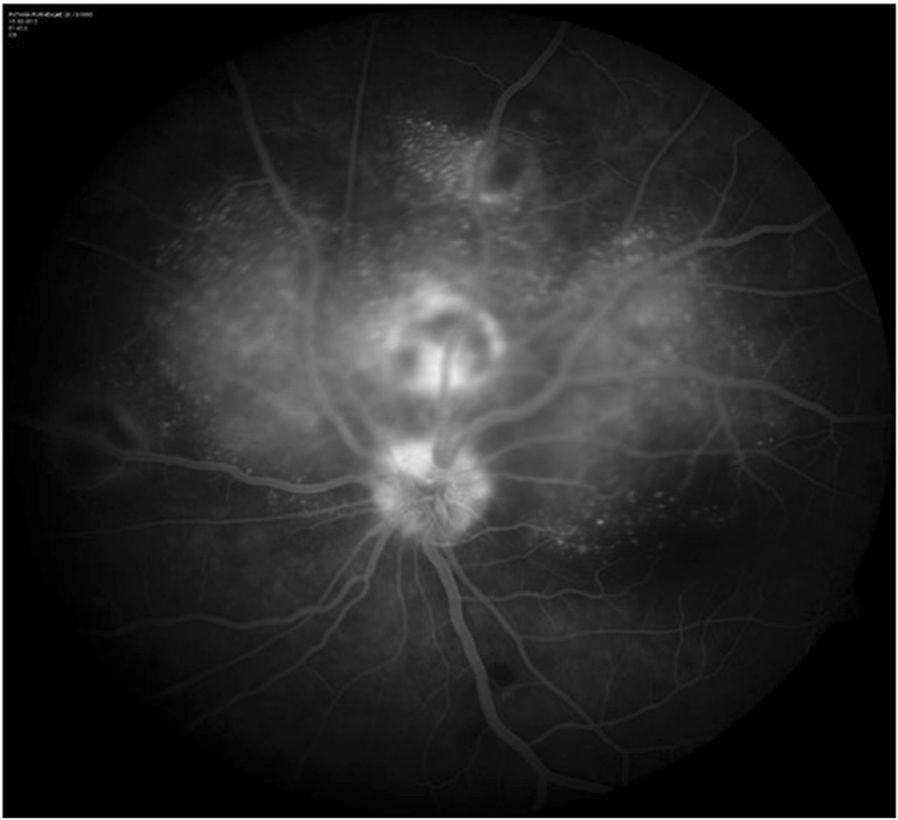
**Late arteriovenous phase of fluorescein angiogram of the left eye showing progressive subretinal pooling of dye.**

**Figure 6:**
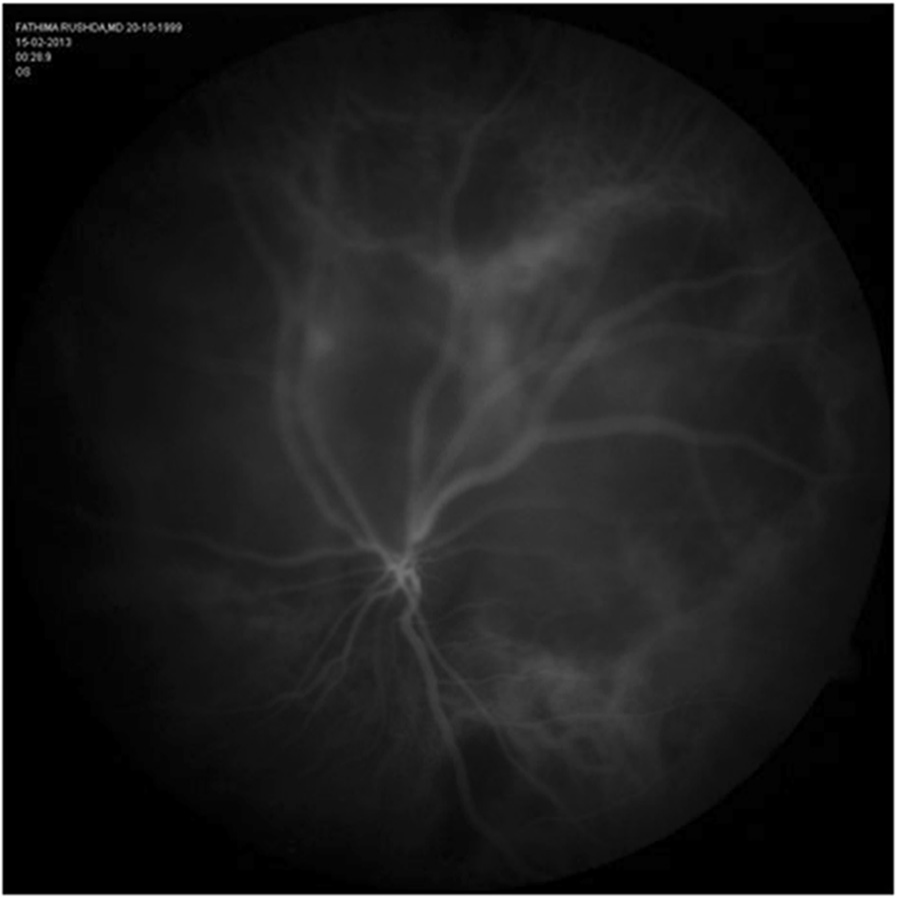
**Early-phase ICG of left eye showing a large hypocyanescent area superior and superotemporal to disc.**

**Figure 7:**
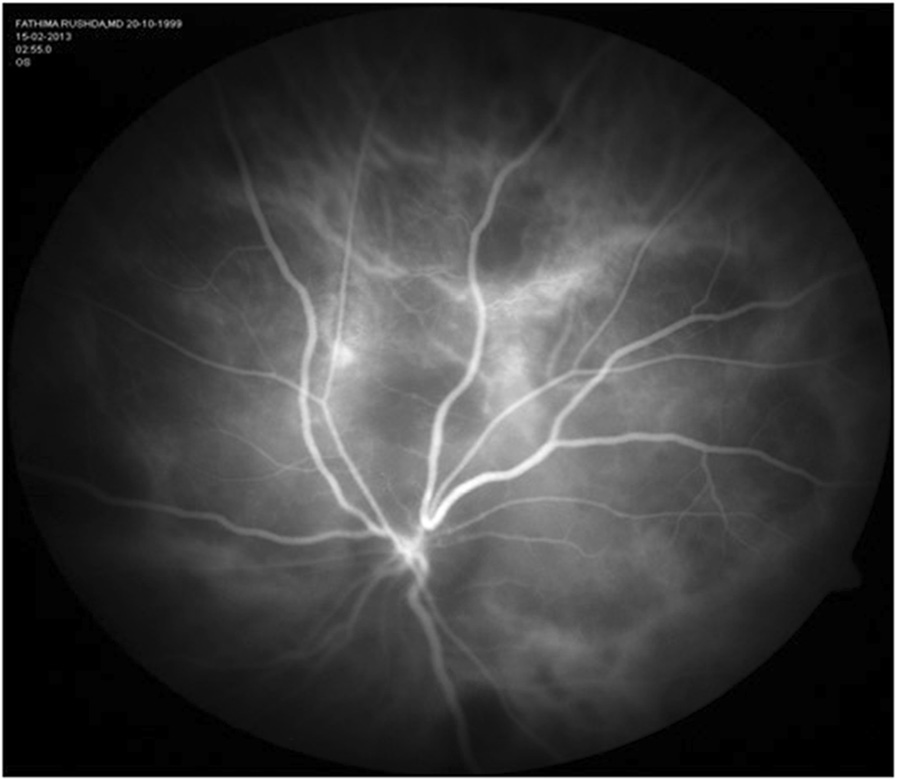
**Late-phase ICG of the left eye showing a large hypocyanescent area superior and superotemporal to the disc.**The area is suggestive of a full-thickness lesion.

**Figure 8:**
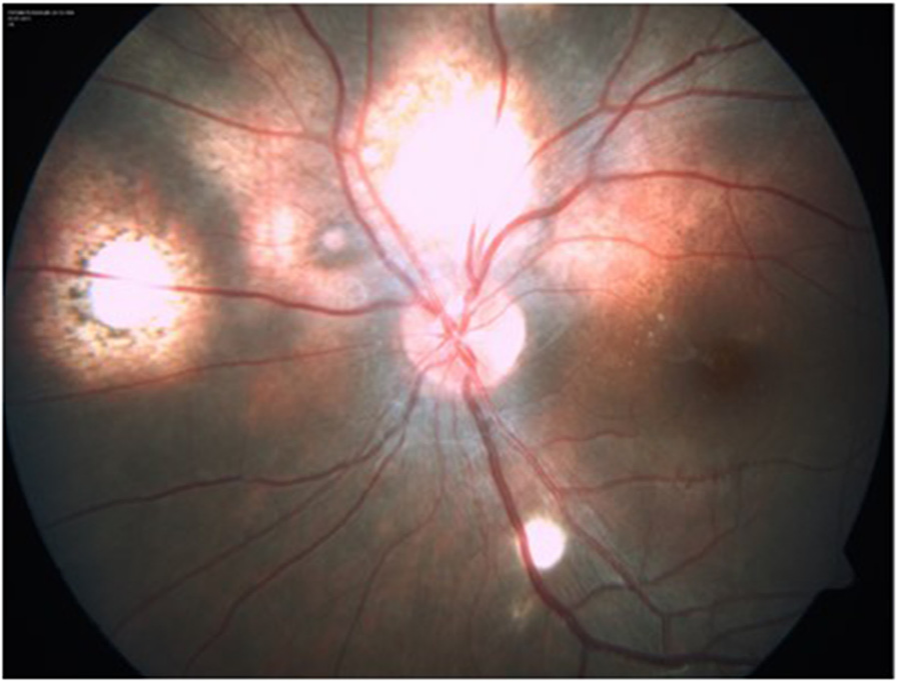
**Clinical photograph of left eye showing healing of lesion following ATT.**

A 13-year-old girl presented with complaints of diminution of vision in her left eye for 3 weeks. On examination, her best corrected visual acuity for distance and near was 6/6 with N6 in her right eye and 6/24 and N6 with effort in her left eye, respectively. Slit lamp examination of the anterior segment and intraocular pressure were normal. Vitreous was normal with no cells or haze. Dilated fundus examination of the right eye revealed multiple, well-defined, yellow subretinal, healed choroidal tuberculomas (Figure 1). The left eye showed a single ill-defined, yellow subretinal lesion with dimensions of 20 × 16 mm located along the superotemporal arcade with surrounding edema and exudative retinal detachment. FFA showed diffuse hyperfluorescence in the early phase (Figures 2 and 3) and pooling of dye in the late phase (Figure 4). ICG showed hypocyanescent lesions that corresponded to the choroidal tuberculomas (Figures 5, 6, and 7). OCT in the left eye showed serous detachment of the neurosensory retina involving the macular area (Figure 8). Systemic evaluation with high-resolution CT showed few small calcified mediastinal lymph nodes and evidence of splenic tuberculosis.

**Figure 9:**
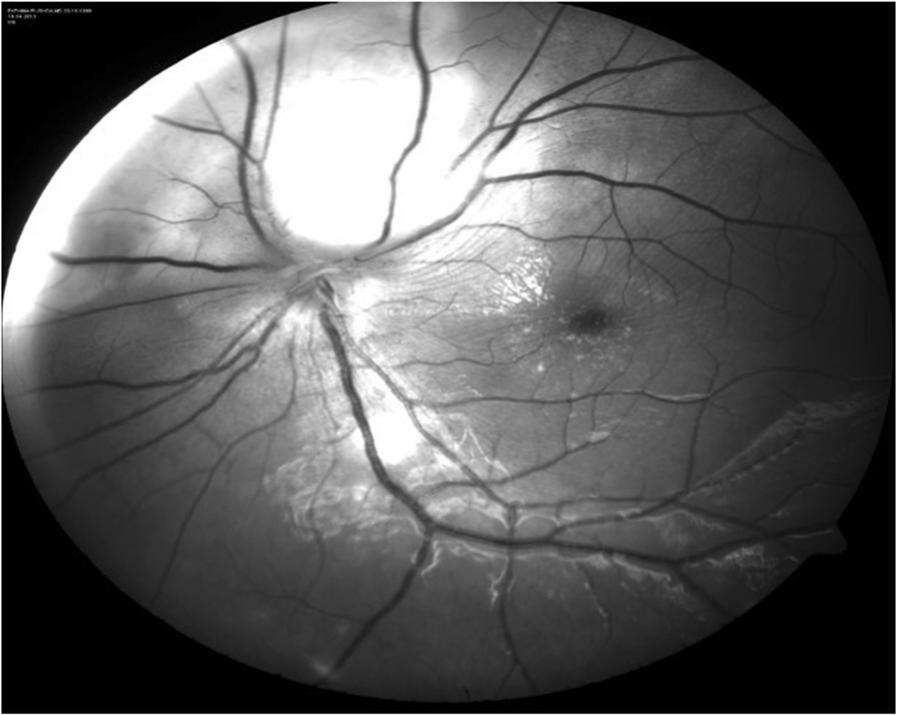
**Complete resolution of the lesion after ATT and oral steroids.**

An anterior chamber tap was performed and the aqueous humor was analyzed by staining for acid-fast bacilli, by polymerase chain reaction (PCR), and by real-time polymerase chain reaction (RT-PCR) kit to detect *Mycobacterium tuberculosis* (MTB) complex genome. DNA extraction from the aqueous humor was carried out using QIAMP DNA extraction kit (Qiagen, Venlo, the Netherlands) and RT-PCR for MTB was carried out using Genosen's MTB complex quantitative real-time PCR kit (Genome Diagnostics, Parwanoo, India). Aqueous humor analysis revealed the absence of acid-fast bacilli, and both PCR and RT-PCR were negative for tuberculosis genome. On the basis of clinical, angiographic, and radiological evidence, multiple healed choroidal tuberculomas in the right eye and active military choroidal tuberculoma in the left eye were diagnosed. Management with anti-tubercular treatment in the form of four drugs with isoniazid, ethambutol, pyrazinamide, and rifampicin for 3 months and isoniazid and rifampicin for 6 months was done. This was supplemented with prednisolone tablet 30 mg a day for 1 week tapered to 5 mg weekly to help resolve the surrounding choroidal inflammation along with ranitidine tablet and oral calcium supplements. On review after 3 months, the patient showed an improvement in her best corrected visual acuity to 6/6 and N6 in the left eye. On examination, the choroidal tuberculoma was found to have completely resolved, the ocular status was stable, and the only evidence of the lesion was pigmentation (Figure 9).

### Discussion

Choroiditis is the most common ocular manifestation in patients with pulmonary and systemic tuberculosis [3]. A history of miliary tuberculosis, clinical examination, angiography, and radiological investigations led to the diagnosis of choroidal tuberculoma in our patient. In the absence of definite evidence, our diagnosis was presumptive and was based on presentation, systemic evaluation, and response to treatment. Choroidal tubercles are usually unilateral but may be solitary or multiple lesions, yellowish with indefinite borders located in the posterior pole, and may vary in their presentation with features such as associated hemorrhage, striae, or exudative retinal detachment [4]. Generally, retinal vessels overlying these lesions appear normal but exudation and hemorrhage have been reported [5]. Choroidal tuberculoma was found to have very little associated inflammation or retinal change in a patient with acquired immunodeficiency syndrome [6]. It has been reported in association with glomerulonephritis [7] and with meningoencephalitis [8]. A case of pulmonary tuberculosis presenting with choroidal granuloma has been reported by Jabbour et al. [9]. They noted an early hyperfluorescence within the lesion that increased in intensity and size over the subsequent phases as the dye leaked into the surrounding serous detachment and late leakage from the lesion. A variation was observed by Milea et al. [10] who described early prolonged blockage and late moderate hyperfluorescence of the tuberculoma. Since there are no typical FFA findings for choroid tuberculoma, and varying appearances may be noted, FFA can contribute to exclusion of other causes. Tayanc et al. [11] reported that ICG showed hypofluorescence of the choroidal tuberculoma in all phases which became less hypoflourescent after treatment and concluded that lesions detected by ICG were larger and more in number than those detected by FFA. Choroidal tuberculoma has been reported in association with AIDS [12], tuberculosis of the spine, and miliary tuberculosis and masquerading as choroidal tumors [13]. Rarely, patients present with ocular signs of isolated presumed choroidal tuberculoma masquerading as an intraocular tumor [14] or choroidal melanoma [15]. There is an increased likelihood of an association with central nervous system tuberculosis, and imaging studies are required in these patients to decide on the duration of anti-tubercular treatment (ATT) [16]. However, a seemingly innocuous ocular involvement could be associated with significant systemic tuberculosis and thus requires thorough investigations. Choroidal tuberculomas can exist in the presence or absence of active disease, and even underlying latent tuberculosis has to be treated with ATT [17]. These tubercles can sometimes remain asymptomatic and may not always incite inflammation but have rarely been reported to be associated with anterior uveitis [18]. In TB granuloma, OCT reveals an area of localized adhesion between the choriocapillaris-RPE layer and overlying the neurosensory retina. This ‘contact’ sign is possibly due to inflammatory adhesions overlying the granuloma, and inflammatory cells appear as increased reflectivity in the deeper retinal layers over the granuloma [19]. Syphilis, toxoplasmosis, sarcoidosis, brucellosis, histoplasmosis, hemangioma, metastasis, foreign body, and amelanotic melanoma are included in the differential diagnosis of choroidal tuberculoma. Histopathologic confirmation, which is needed for definite diagnosis of choroidal tuberculoma, is not practical; therefore, diagnosis of choroidal tuberculoma is usually presumptive and is based on clinical and laboratory findings [20]. Isolated uveal tuberculoma can present in an otherwise healthy patient with negative systemic tuberculosis evaluation, and PCR can be used to confirm tuberculosis when other methods have failed [21]. A vitreous or fine needle aspiration biopsy may be useful in clinching the etiological diagnosis in difficult situations. A high degree of clinical suspicion is important, especially in migrants from developing countries. Molecular diagnosis of tuberculosis by polymerase chain reaction is being increasingly used for diagnosis [22]. Apart from angiography, detection of DNA and quantification of the mycobacterial load by RT-PCR in intraocular fluids is a development which can complement existing investigations in precise identification [23].

### Conclusion

A choroidal tuberculoma is one of the most common forms of ocular tuberculosis, and it is probably due to high blood supply of the choroid. This case highlights the fact that in some patients, analysis of the aqueous may not provide any clue to the confirmation of tubercular choroidal granuloma. An association between splenic tuberculosis and choroidal tubercles has not been reported till now. This patient was not very symptomatic despite chest and spleen involvement, thus proving that miliary tuberculosis has various forms of presentation. After the diagnosis of presumed or confirmed tuberculosis is established, surgical intervention should be avoided. Detection of choroidal inflammation can prevent visual loss as the ocular lesion resolves fully with timely management. Miliary tuberculosis was found as one of the most important independent factors linked to the development of ocular tuberculosis [24]. Since delay in diagnosis and treatment results in poor prognosis and severe sequelae, effective therapy should be initiated as early as possible. Response to the treatment may be helpful to confirm the diagnosis of choroidal tuberculoma.

## Abbreviations

ATT: anti-tubercular treatment
DNA: deoxyribonucleic acid
FFA: fundus fluorescein angiography
ICG: indocyanine green angiography
MTB: *Mycobacterium tuberculosis*
OCT: optical coherence tomography
PCR: polymerase chain reaction
RT-PCR: real-time polymerase chain reaction
TB: ocular tuberculosis
